# The Impact of Silicon Nanoparticle Porosity on Their Ability to Sensitize Low-Intensity Medical Ultrasound

**DOI:** 10.17691/stm2025.17.1.04

**Published:** 2025-02-28

**Authors:** L.A. Osminkina, P.A. Tyurin-Kuzmin, M.V. Sumarokova, A.A. Kudryavtsev

**Affiliations:** PhD, Leading Researcher, Head of the Laboratory of Physical Methods, Biosensorics and Nanotheranostics, Medical Physics Department, Faculty of Physics; Lomonosov Moscow State University, 1 Leninskiye Gory, Moscow, 119991, Russia; Senior Researcher; Institute for Biological Instrumentation of the Russian Academy of Sciences, 7 Institutskaya St., Pushchino, 142290, Russia; DSc, Associate Professor, Faculty of Fundamental Medicine; Lomonosov Moscow State University, 1 Leninskiye Gory, Moscow, 119991, Russia; Senior Researcher; Institute for Biological Instrumentation of the Russian Academy of Sciences, 7 Institutskaya St., Pushchino, 142290, Russia; Student, Faculty of Physics; Lomonosov Moscow State University, 1 Leninskiye Gory, Moscow, 119991, Russia; PhD, Leading Researcher; Institute of Theoretical and Experimental Biophysics of the Russian Academy of Sciences, 3 Institutskaya St., Pushchino, 142290, Russia; Senior Researcher; Institute for Biological Instrumentation of the Russian Academy of Sciences, 7 Institutskaya St., Pushchino, 142290, Russia

**Keywords:** sonodynamic therapy, silicon nanoparticles, sensitization, ultrasound, cavitation, biocompatibility

## Abstract

This study investigates the role of porosity in silicon nanoparticles’ ability to act as sonosensitizers for sonodynamic therapy of malignant tumors.

Structural analysis showed that porous nanoparticles are composed of nanocrystals approximately 4 nm in size and contain 15 nm pores, whereas non-porous nanoparticles have a dense structure with nanocrystals ranging from 10 to 50 nm. Porous nanoparticles exhibit pronounced photoluminescent properties, associated with quantum confinement effects in their small nanocrystals.

The cytotoxicity of the nanoparticles was investigated in vitro using Hep2 cells. The results showed that both porous and non-porous nanoparticles in the studied concentration range (2–500 μg/ml) are non-toxic. Low-intensity ultrasound (0.88 MHz, <1 W) also does not have a toxic effect on the cells. However, the combined use of porous nanoparticles and ultrasound led to a significant decrease in cell viability, which was not observed when non-porous nanoparticles were used. This effect is associated with mechanical destruction of the cell membranes, as well as the potential activation of additional cell death mechanisms, such as apoptosis.

The results highlight the importance of porosity as a key factor determining the effectiveness of silicon nanoparticles as sonosensitizers. The high efficiency, low toxicity, and unique structural properties of porous nanoparticles make them a promising material for further research and development of targeted, non-invasive treatments for malignant tumors in the context of sonodynamic therapy.

## Introduction

Sonodynamic therapy (SDT) is a method to treat malignant tumors combining the effect of low-intensity ultrasound using sonosensitizers — agents enhancing the sensitivity of tumor cells to ultrasound radiation [1]. In recent years, special attention has been paid to the use of nanoparticles as sonosensitizers, it opening up new prospects in oncologic therapy.

Nanoparticles due to their unique physicochemical properties can enhance the therapeutic effect of ultrasound through several mechanisms. Firstly, they contribute to the generation of reactive oxygen species in ultrasound radiation resulting in an oxidative stress and the following death of tumor cells. Secondly, nanoparticles can serve as cavitation nucleation centers reducing the cavitation threshold and enhancing the mechanical breakdown of cell structures. Thirdly, some nanoparticles effectively absorb ultrasound energy converting it into heat and causing local hyperthermia leading to tumor tissue destruction [2–4].

SDT using nanoparticles also enables to achieve high effect selectivity. Due to possible surface functionalization, nanoparticles can be directed into tumor cells minimizing the damage of intact tissues. However, despite major advances in the sphere, the development of effective and safe nanoparticlessonosensitizers is still a critical task that needs further studies and clinical trials [4, 5].

Currently, porous silicon nanoparticles are of particular interest, they have unique characteristics: biocompatibility, ability to biodegrade to nontoxic silicic acid, as well as photoluminescence (PL) in the visible spectrum due to quantum-confinement effects [6]. The mentioned properties make porous silicon nanoparticles a universal material for targeted drug delivery and their noninvasive monitoring. It is important to emphasize that the nanoparticle size and porosity can be varied to adapt them to certain medical challenges [7, 8]. The studies demonstrated porous silicon nanoparticles to have low toxicity both *in vitro* and when administered intravenously *in vivo*, and be effectively absorbed by tumor cells that makes them safe and promising to be used in biomedicine [6, 9].

Porous silicon nanoparticles are considered as promising sonosensitizers for SDT. One of their key properties is the ability to play the role of nucleation centers of cavitation bubbles that significantly reduces the incipient cavitation threshold under ultrasound [10, 11]. Cavitation characterized by the formation and the following collapse of microbubbles in liquid medium causes the mechanical destruction of cellular membranes and, as a result, the death of tumor cells.

At a pre-cavitation stage, i.e. before the complete cavitation, silicon nanoparticles also have a major effect on cell structures. The study [12] showed that the combined effect of nanoparticles and ultrasound can induce apoptosis — programmed cell death (it promotes tumor cell elimination with no inflammatory response following). Thus, the combined usage of ultrasound and nanoparticles improves overall therapy efficiency due to a synergistic effect on tumor tissue. The data were proved by *in vitro* and *in vivo* experimental findings demonstrating high efficiency of porous silicon nanoparticles as sensitizers for tumor ultrasound therapy [10–14].

**The present study aimed** at investigating the effect of porous silicon nanoparticles on their ability to reduce the thresholds of acoustic cavitation and enhance a therapeutic effect of low-intensity ultrasound. To achieve the objective we studied structural, optical, and sonosensitizing properties of nanoparticles, as well as their impact on *in vitro* cell viability.

## Materials and Methods

### Nanoparticle synthesis

Aqueous suspensions of porous (pSi-NPs) and non-porous (Si-NPs) silicon nanoparticles were obtained by crushing porous and non-porous silicon nanowire arrays, respectively.

Silicon nanowire arrays were obtained using metalassisted chemical etching (MACE). At the first stage the с-Si (100) substrate was placed into the mixture of 0.01 М AgNO_3_ and 5 М HF, their volume ratio being 1:1, for 15 s. The process resulted in depositing Ag nanoparticles on c-Si surface. Then the c-Si substrate was placed into the mixture of 5 М HF and 30% H_2_O_2_, the volume ratio being 10:1, where the process of chemical etching started. To remove Ag nanoparticles, the samples were placed into 35% HNO_3_ for 15 min. After that the samples were washed 3 times in distilled water (Merck Millipore, Germany) and air-dried at room temperature.

Porous silicon nanowires were obtained using MACE of heavily boron-doped c-Si with resistivity 0.001 Ohm·cm. Non-porous silicon nanowires were produced via MACE of lightly boron-doped c-Si with resistivity 1 Ohm·cm [15].

The obtained silicon nanowires were mechanically separated from c-Si substrates and fragmented in distilled water (Merck Millipore, Germany) in an ultrasonic bath (Elmasonic, Germany; 37 kHz) for 12 h. After ultrasound exposure, the resulted nanoparticle solutions sedimentated for 24 h, and centrifuged within 5 min at 1300 bpm to remove large uncrushed nanowires; the obtained supernatant was used in the experiment. The grinding of porous and non-porous nanowires resulted in obtaining the suspensions of pSi- NPs and Si-NPs, respectively.

### Characterization of nanoparticles

The obtained samples were structurally studied using a transmission electron microscope (LEO 912 AB OMEGA; Carl Zeiss, Germany). Malvern Zetasizer Nano ZS (Malvern Instruments Ltd, Great Britain) was used to determine the size and zeta-potential (ZP) of silicon nanoparticles according to dynamic light scattering (DLS). The composition of the sample surface was studied using IR-Fourier spectrometer IR-8000 (САС, Russia) with an attenuated total reflection (ATR). The Raman scattering (RS) spectra of were measured using a confocal Raman microscope ConfotecTM MR350 (SOL instruments, Belarus Republic). For Raman measurements, an excitation wavelength 633 nm and low laser power 1 mW to protect the samples against overheating. Before Raman measuring, 20 μl of nanoparticle suspension was applied on the plate of crystal Ge, and air-dried.

### In vitro study of nanoparticle toxicity

Hep2 cell line (human laryngeal carcinoma) were cultured in DMEM medium containing 10% fetal bovine serum (Gibco, USA), L-glutamine (600 mg/L), HEPES (20 mM), and gentamicin (80 mg/L). The incubation was carried out at 5% СО_2_ and 37°C. For experiments the cells were cultured in 12-well plates at concentration of 100,000 cells per well, with culture medium, 1 ml per well.

The required amount of silicon nanoparticles was added 24 h after seeding the cells. Before addition, silicon nanoparticles were centrifuged into the culture medium and exposed to ultrasound in an ultrasound bath, with frequency of 30 kHz with pipetting for regular distribution of nanoparticles in the solution. The tube with nanoparticles was placed in the cavitation zone for maximum nanoparticle dispersibility.

24 h after adding the nanoparticles, the cells were washed to remove unbound nanoparticles. The number of cells in each well was determined by calculating in a hemocytometer. To assess the phase composition of the cells after nanoparticle exposure, the samples were stained by propidium iodide (50 μg/ml) followed by analyzing the samples on a flow cytofluorometer PASIII (Partec, Germany). It enabled to determine of the proportion of viable and dead cells, as well as the distribution across the cell cycle phases.

### The study of the combined effect of nanoparticles and ultrasound on cells in vitro

Hep2 line cells were cultured in culture flasks (surface area 25 cm^2^) within 72 h. The culture medium DMEМ contained 10% fetal bovine serum (Gibco, USA), L-glutamine (600 mg/L), HEPES (20 mM), and gentamicin (80 mg/L). The cells were incubated at 37°C and 5% CO_2_. The flask had 5 ml of the culture medium.

The samples of aqueous nanoparticle suspensions were centrifuged, dried in a drying chamber to remove residual water, followed by grinding in an agate mortar for 5 min, and adding the cultural medium to get a homogeneous suspension. The cell culture medium was substituted by the medium containing nanoparticles, and incubated within 2–4 h. The nanoparticle concentration was 300 μg/ml. After incubation the cells were washed 3 times using Hank’s solution taken from the flask by trypsinization, and transformed into standard Hank’s solution. The cell number was regulated up to the concentration of 2×10^5^ cells/ml.

The prepared cell suspensions were exposed to ultrasound using the device for ultrasound therapy UZT- 1.3.01F-Med TeKo (Med TeKo, Russia). Ultrasonic frequency was 0.88 MHz, power output — 1 W, modulation — 2/20. The experiment was carried out using polypropylene tubes (Greiner Bio-One, Austria; 50 ml, diameter — 29 mm, with a conical bottom, the cone height — 18 mm) containing the suspension of cells with nanoparticles, 1 ml. Degassed distilled water (at 37°C) was used as a contact medium between a flat ultrasonic transducer with a radius of 2 cm and a cuvette filled with the sample. The tubes were immersed in water to a depth of 15–18 mm, with the bottom of the tube positioned 66 mm above the surface of the ultrasonic transducer located at the bottom of the bath. In the control group, cells were studied without silicon nanoparticles.

Live and dead cells were distinguished using trypan blue staining (Gibco, USA) (0.4%, 1:1). The counting of live cells was performed using a hemocytometer, with measurement error assessed using point and interval analysis at a confidence level of 0.95.

### Optical holotomography

The experiments on bioimaging *in vitro* were carried out using a holotomographic microscope HT-X1 (TomoCube, Republic of Korea), by courtesy of Bioline Company (Russia). The device had a fluorescent block with a diode source of blue light, an air objective with 40- fold increase, and CMOS camera with the resolution 2.8 megapixels. To maintain the cells viability we used the desk-top incubator, where we maintained the temperature 37°С, humidity 100%, gas composition — 5% СО_2_. The cells were cultured on confocal dishes with a thin bottom (0.17 mm) in the evening before the experiment for complete spreading. The nanoparticles were added to the cells 2 h prior to the imaging beginning. The imaging was performed in the complete cell growth medium.

## Results

### The study of morphology and physicochemical properties of nanoparticles

**Figure 1.**
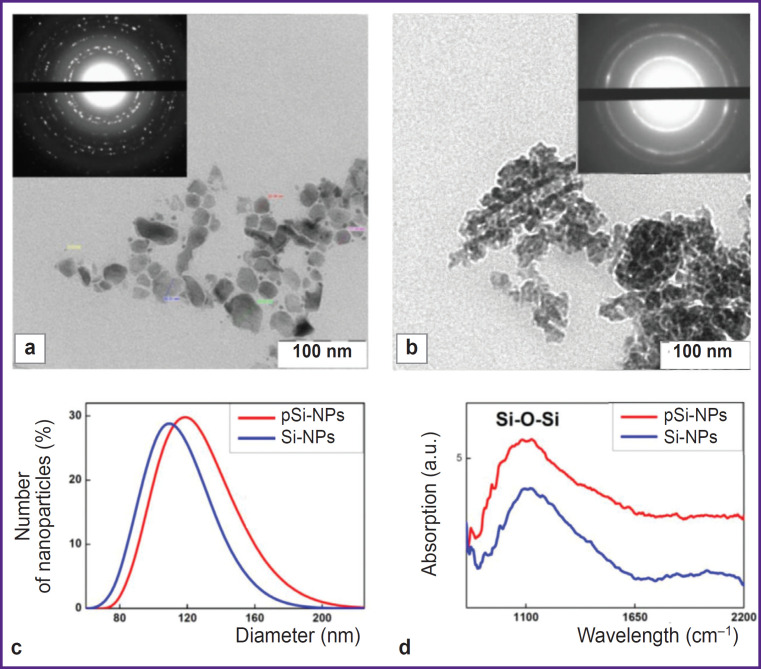
Silicon nanoparticles characterization: (а), (b) TEM micrographs of nonporous Si-NPs and porous pSi- NPs, respectively, obtained by transmission electron microscopy; electron diffraction patterns are shown in the insets; (c) nanoparticle size distribution obtained by dynamic light scattering; (d) FTIR absorption spectra of silicon nanoparticles

The structural properties of Si-NPs and pSi-NPs obtained through ultrasound fragmentation of silicon nanowire arrays were investigated using transmission electron microscopy (TEM) and DLS. TEM microphotographs (Figure 1 (a), (b)) demonstrated that both types of nanoparticles were 50–150 nm in size, which is consistent with the results obtained by DLS (Figure 1 (c)). At the same time, Si-NPs have a non-porous structure, while pSi-NPs consisted of small nanocrystals and ~15 nm pores.

ZP measurements showed negative values: –25 mV for Si-NPs, and –26 mV for pSi-NPs. These values give evidence of stable colloid systems and no tendency for particle aggregation in the suspension.

The electron diffraction patterns, shown in the insets of the TEM microphotographs (see Figure 1 (a), (b)) revealed significant differences between Si-NPs and pSi- NPs. Si-NPs are characterized by strongly pronounced isolated narrow diffraction peaks that suggests the presence of the small amount of randomly oriented silicon nanocrystals (relatively large, >10 nm). In contrast to them, pSi-NPs demonstrate wide concentric rings corresponding to a large number of tiny (<5 nm) silicon nanocrystals. This confirms the porous structure of pSi-NP, where nanoparticles consist of numerous tiny nanocrystals.

Infrared transmission spectra (Figure 1 (d)) showed a prominent broad band at 1100 cm^–1^, corresponding to Si- O-Si bonds, indicating the presence of an oxide layer on the surface of the silicon nanocrystals. The formation of the oxide layer is attributed to the treatment of nanowire arrays in HNO_3_ to remove silver particles after the MACE process, as well as the mechanical fragmentation of nanowires into nanoparticles in water. It should be noted that the presence of an oxide layer enhances the dispersibility of nanoparticles in an aqueous medium due to the hydrophilic properties of their surface.

**Figure 2.**
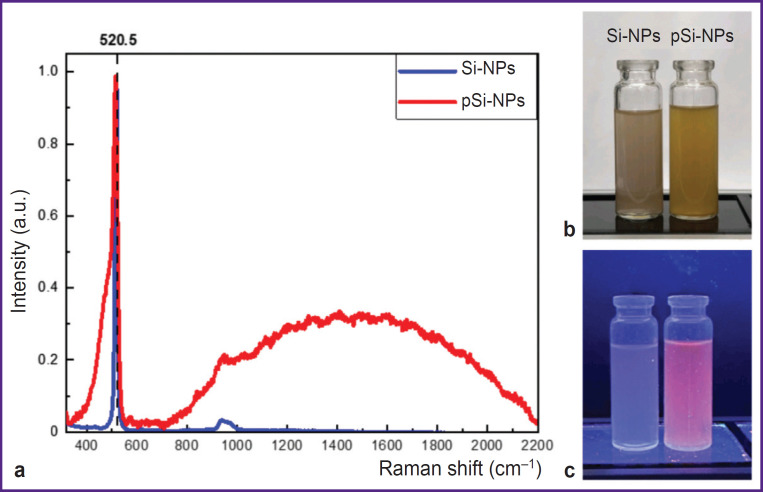
Raman scattering spectra for non-porous Si- NPs and porous pSi-NP (a); suspensions of porous and nonporous silicon nanoparticles under normal lighting (b); the same suspensions under ultraviolet lighting, showing photoluminescence (c)

Figure 2 (а) demonstrates RS spectra for the Si- NPs and pSi-NPs samples. The spectra represent the scattering line typical for nanocrystalline silicon; with its maximum shifted by Δω relative to 520.5 cm^–1^. This corresponds to the transverse optical (TO) phonon vibrations in crystalline silicon (c-Si). The position of the Raman scattering peak is indicated by a dashed line. The low-frequency shift of the Raman peak in the nanoparticle spectra is associated with the quantum confinement of phonons in small nanocrystals. The nanocrystal diameters, calculated from the peak position [16], were 10 nm for Si-NPs and 4.3 nm for pSi-NPs. This confirms the reduction in nanocrystal size in porous samples, leading to more pronounced quantum effects related to phonon confinement.

The spectra of porous silicon nanoparticles exhibit a broad peak at 480 cm^–1^, which is characteristic of the amorphous silicon phase. The partial amorphization of nanocrystals is likely a result of the fabrication process and storage of the samples in water. Additionally, the spectrum of porous samples shows a PL peak with a maximum around 700 nm. The observed PL originates from the radiative recombination of excitons formed in small silicon nanocrystals upon photoexcitation. The broad PL spectrum is attributed to the size distribution of the nanocrystals [15].

The photos of nanoparticle suspensions in distilled water (Figure 2 (b), (c)) illustrate visual differences between porous and non-porous samples. The image (b) shows the suspensions at normal lighting. pSi-NP suspension had the marked tint of yellow compared to Si-NP suspension, which can be attributed to the higher porosity and larger surface area of the nanoparticles [17]. Under ultraviolet illumination (see Figure 2 (c)) pSi-NPs exhibit PL in the red spectral region, whereas Si-NP suspension emits more weakly and has a bluish tint.

### Cytotoxicity assessment of nanoparticles in vitro

**Figure 3.**
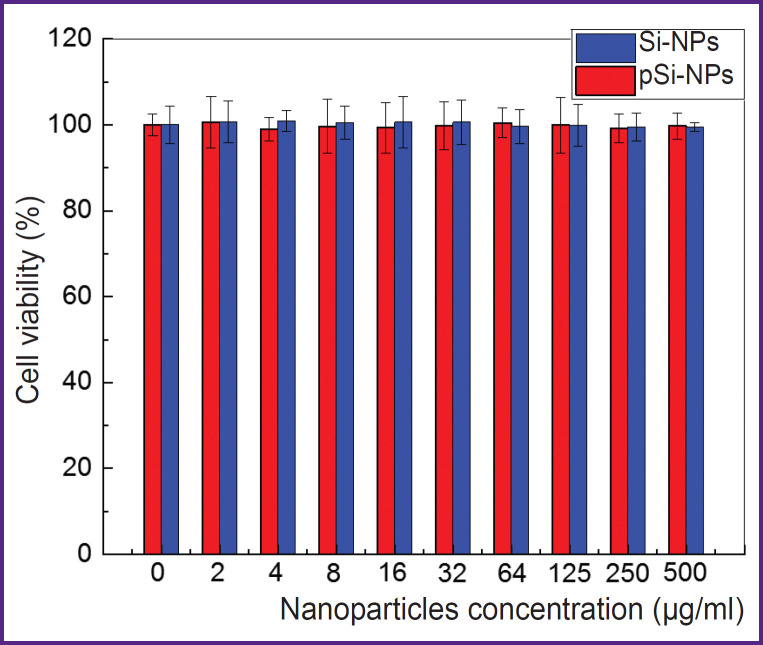
Cell viability of Hep2 cells as a function of non-porous Si-NPs and porous pSi-NP concentrations relative to the control group without nanoparticles

Figure 3 presents the dependence of cell viability on the concentration of Si-NPs and pSi-NPs *in vitro*.

According to the findings, the cell survival was close to 100% for all nanoparticle concentrations in the range from 2 to 500 μg/ml. It suggests both Si-NPs and pSi-NPs in the mentioned concentrations have no pronounced cytotoxic effect on Hep2 cells.

### The assessment of a combined effect of lowfrequency ultrasound and nanoparticles on cells in vitro

**Figure 4.**
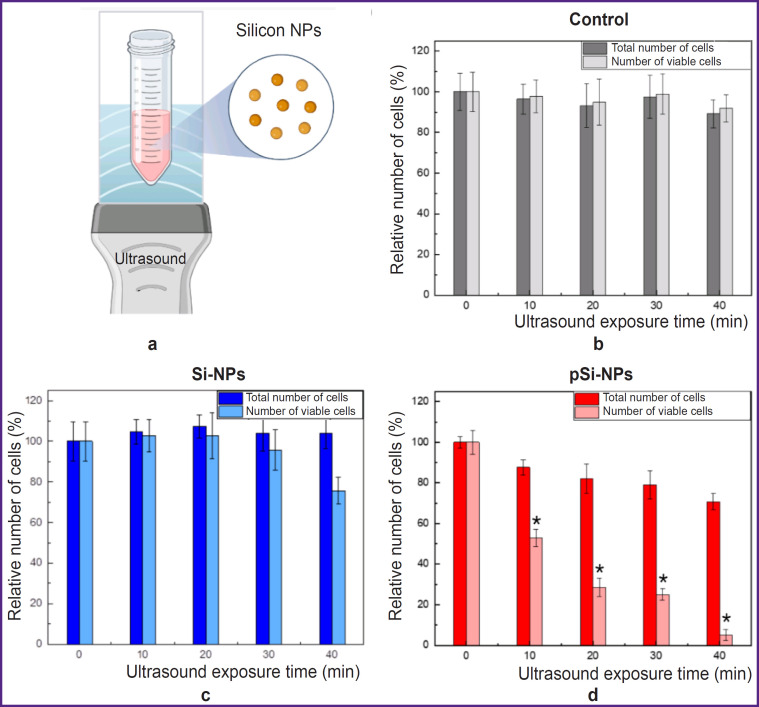
Effect of low-intensity ultrasound and silicon nanoparticles on Hep2 cell viability *in vitro*: (a) schematic representation of the experimental setup with ultrasound exposure; (b) dependence of the total number of cells and the number of viable Hep2 cells on time under ultrasound exposure; (c) dependence of the total number of cells and the number of viable Hep2 cells on time under ultrasound exposure and non-porous Si-NPs; (d) dependence of the total number of cells and the number of viable Hep2 cells on time under ultrasound exposure and porous pSi-NP; * p<0.05

Figure 4 demonstrates the experimental findings of a combined effect of low-intensity ultrasound and silicon nanoparticles with varying porosity on Hep2 cells *in vitro*.

Figure 4 (а) shows the scheme of the experimental setup, with a detailed description provided in section “Materials and Methods”. Figure 4 (b) illustrates the data for the control group of Hep2 cells exposed to ultrasound. The total number of cells and the number of viable cells remain virtually unchanged at all time intervals of ultrasound exposure (0, 10, 20, 30, and 40 min). This indicates that in the selected experimental conditions, low-intensity ultrasound exposure at 0.88 MHz and power <1 W has no significant effect on the cells.

In a combined effect of ultrasound and Si-NPs at a non-toxic concentration (300 μg/ml) there was a slight decrease of the total cell count and the number of viable cells with increasing ultrasound exposure time. This indicates the low cytotoxicity of Si-NPs when activated by ultrasound (Figure 4 (c)).

Figure 4 (d) demonstrates the results for the cells under a combined effect of ultrasound and pSi-NPs at a non-toxic concentration (300 μg/ml). It is evident that with increasing ultrasound exposure time, there is a significant decrease in the number of viable cells, indicating the high cytotoxicity of pSi-NPs when activated by ultrasound.

Porous silicon nanoparticles proved to be more effective sonosensitizers when exposed to ultrasound compared with non-porous nanoparticles, due to key features of their structure. The porous structure of nanoparticles contributes to retaining dissolved gas molecules within the pores, it resulting in the formation of nucleation centers significantly lower the cavitation threshold during ultrasound exposure [10]. Thus, even if the ultrasound amplitude is insufficient to cause complete cavitation, pSi-NPs are able to locally enhance cavitation processes. Cavitation is accompanied by forming bubbles, which when collapsed can create local areas of high pressure and temperature. Such extreme conditions can cause mechanical destruction of cell structures, membrane damage, or cell lysis, which explains the decrease in the total number of cells in this experiment.

Additionally, the decrease in the number of viable cells upon ultrasound exposure with porous nanoparticles suggests the involvement of more complex cell death mechanisms, such as apoptosis. In this case, even in the absence of visible macroscopic cavitation, porous nanoparticles may act as cavitation nucleators, increasing the likelihood of local cavitational events. This, in turn, enhances ultrasound-induced cytotoxicity through a combination of mechanical disruption and activation of biochemical pathways leading to cell death.

### The study of interaction of nanoparticles and cells in vitro using optical holotomography

**Figure 5.**
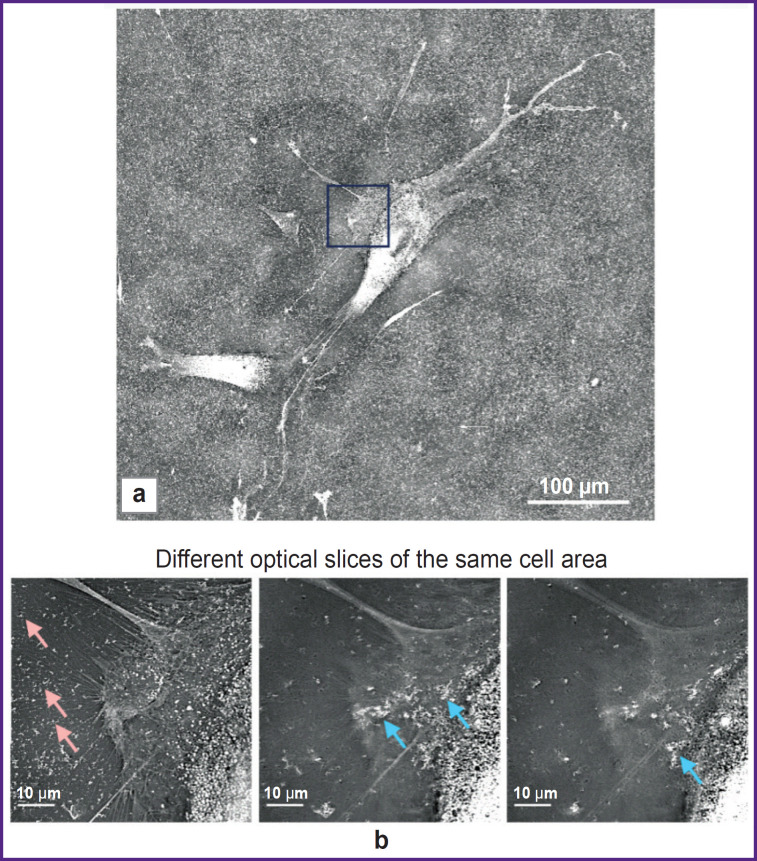
Holotomographic images of the cells incubated with porous silicon nanoparticles: (a) general view of the cell; the area marked with a square indicates the region, with optical slices shown in (b); (b) optical slices taken at different heights from the substrate reveal that, two hours after adding nanoparticles to the cells, most of them do not enter the cell but instead adhere to the plasma membrane. In some areas, nanoparticles accumulate into aggregates on the cell surface (*blue arrow*), in contrast to extracellular regions where the nanoparticles are distributed relatively evenly (*pink arrow*)

According to the data represented above, a key mechanism of a toxic effect of ultrasound activated by pSi-NPs is the impact on cellular membranes. Consequently, an important element of a damaging pSi-NPs effect under ultrasound is their placement in close proximity to the cell membranes. Holotomographic microscopy was used to check pSi-NPs position among subcellular membrane structures. To examine how pSi-NPs are positioned among subcellular membrane structures, we employed holotomographic microscopy. This method, by irradiating cells in the visible spectrum, allows the visualization of membrane structures based on differences in the refractive index between the aqueous and lipid phases. As shown in Figure 5, nanoparticles adhere to the cell membrane at a concentration significantly higher than that in the aqueous phase of the extracellular environment. Therefore, upon ultrasound exposure, the damaging effect on the cell membrane will be intensified.

## Conclusion

The present study investigated structural, optical, and sonosensitizing properties of porous and nonporous silicon nanoparticles in order to apply them in sonodynamic therapy of malignant tumors. The obtained data indicate that porous silicon nanoparticles possess unique properties that significantly enhance their effectiveness as sonosensitizers.

Structural analysis results revealed significant differences between porous and non-porous silicon nanoparticles. Porous nanoparticles are composed of nanocrystals approximately 4 nm in size and have a porous structure with pores about 15 nm in diameter. In the electron diffraction patterns of porous nanoparticles, broadened concentric rings were observed, indicating a high degree of amorphization and the presence of numerous small nanocrystals. In the Raman scattering spectra, a low-frequency shift of the scattering line for porous nanoparticles was detected, which is associated with the quantum confinement of phonons in small nanocrystals. Additionally, the spectra showed a broad peak around 480 cm^–1^, characteristic of the amorphous phase of silicon. In contrast, non-porous nanoparticles have a dense structure and consist of nanocrystals ranging from 10 to 50 nm in size. Their electron diffraction patterns exhibit narrow, isolated peaks, indicating the presence of a greater number of larger, well-ordered nanocrystals. In the Raman spectra for non-porous nanoparticles, the scattering line is positioned closer to the value for crystalline silicon (520.5 cm^–1^), further confirming their higher crystallinity. These data highlight significant structural differences between the two types of nanoparticles, which account for their unique physicochemical properties and potential biomedical applications.

*In vitro* experiments confirmed that both types of nanoparticles do not exhibit significant toxicity to Hep2 cells at the studied concentrations (2–500 μg/ml). Furthermore, the low-intensity ultrasound exposure (0.88 MHz, <1 W), applied in the experiment, also does not have toxic effects on the cells in the absence of nanoparticles. When ultrasound was combined with non-porous nanoparticles, only a slight decrease in cell viability was observed, indicating their limited ability to sensitize ultrasound effects. However, the combination of ultrasound with porous nanoparticles led to a significant decrease in cell viability. Porous nanoparticles not only enhance mechanical cell destruction through the sensitization of localized cavitation processes upon ultrasound exposure but also likely initiate additional cell death mechanisms, including apoptosis.

Thus, the results underscore the importance of porosity as a key structural parameter for enhancing the effectiveness of silicon nanoparticles in sonodynamic therapy. The high efficiency, low toxicity of the nanoparticles, and the absence of direct toxic effects from ultrasound make this combination a promising approach for developing targeted and non-invasive methods for the treatment of malignant tumors. Further studies should focus on exploring the biochemical mechanisms of cell death and optimizing ultrasound exposure parameters for clinical applications.
